# Pancreatic acinar cell metaplasia in the stomach of a 12-year-old child: An extremely rare case report

**DOI:** 10.1097/MD.0000000000043591

**Published:** 2025-07-25

**Authors:** Muhsen Issa, Wafaa Taleb, Ali Ibrahim, Rana Issa

**Affiliations:** a Faculty of Medicine, Al-Andalus University for Medical Sciences, Tartus, Syria; b Cancer Research Center, Tishreen University, Lattakia, Syria; c Department of Pediatrics, Tishreen University Hospital, Lattakia, Syria; d Department of Pathology, Tishreen University Hospital, Lattakia, Syria.

**Keywords:** abdominal pain, case report, Helicobacter pylori, metaplasia, pancreas, stomach

## Abstract

**Rationale:**

Pancreatic acinar cell metaplasia of the gastric mucosa is characterized by the presence of clusters of pancreatic-like cells in the stomach. Although there are many instances of this condition in adults described in the medical literature, symptomatic cases in children are exceedingly rare.

**Patient concerns:**

A 12-year-old female patient presented to the pediatric clinic at Tishreen University Hospital complaining of postprandial nausea and recurrent periumbilical pain. The patient’s symptoms were not accompanied by fever, diarrhea, or constipation. The patient’s medical history was unremarkable. No abnormalities were noted on the physical examination.

**Diagnoses:**

Biopsies taken during esophagogastroduodenoscopy revealed Helicobacter pylori-positive chronic gastritis.

**Interventions:**

The patient was started on omeprazole, clarithromycin, and metronidazole triple therapy, but with minimal improvement. Six months later, esophagogastroduodenoscopy was repeated because her symptoms persisted, and antral biopsies showed clusters of pancreatic-like cells with no signs of H. pylori.

**Outcomes:**

The patient was diagnosed with pancreatic acinar cell metaplasia and put on symptomatic treatment, to no avail. One year after the initial diagnosis, esophagogastroduodenoscopy was redone, showing similar findings.

**Lessons:**

This report shows the development of pancreatic acinar cell metaplasia in a child after treatment for H. pylori. The clinical significance of pancreatic acinar cell metaplasia is not yet known because of the small number of cases documented in the literature, particularly among children.

## 1. Introduction

Pancreatic acinar cell metaplasia (PACM) of the gastric mucosa is characterized by the presence of clusters of exocrine pancreas-like cells in the stomach.^[1]^ PACM was first described in adults in 1993 by Doglioni et al and went unnoticed in children until 1997, when it was reported by Krishnamurthy et al.^[1,2]^ This condition differs from pancreatic heterotropia, as the latter is capable of forming islets of Langerhans and producing pancreatic hormones.^[1]^ In adults, it is frequently associated with Helicobacter pylori infection and chronic atrophic gastritis, while in children, there are no clear associations, as cases among children are exceedingly rare in the literature.^[2,3]^ The etiology of the metaplasia remains unclear, but multiple mechanisms have been implicated in the transformation process.^[4]^ PACM is mostly an incidental finding during esophagogastroduodenoscopy (EGD), and its clinical significance is yet to be determined.^[1]^ There is a lack of articles in the literature discussing the importance and clinical implications of PACM, particularly among children. This case report documents the presence of PACM in a 12-year-old child treated for H. pylori infection.

## 2. Case presentation

A 12-year-old female patient presented to the pediatric clinic at Tishreen University Hospital complaining of postprandial nausea, which was partially responsive to antiemetics, and recurrent periumbilical pain that was slightly mitigated by analgesics and antispasmodics. The symptoms were not accompanied by fever, diarrhea, or constipation. The patient weighed 34 kg, measured 144 cm, and was within the normal range for her age. Her medical history was unremarkable, and no abnormalities were noted on physical examination.

**Figure 1.**
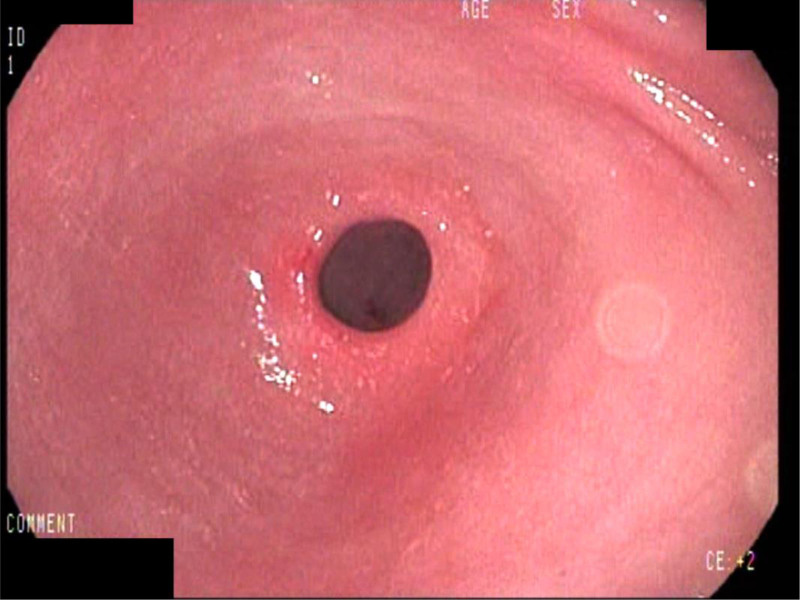
Mild congestion in the antrum and near the pylorus.

Complete laboratory evaluation was performed on the patient and is shown in Table 1. Her complete blood count was within normal limits, and her liver enzyme levels were not elevated. In addition, stool sample analysis, along with a fetal calprotectin test, was normal. Ultrasound assessment of the patient’s abdomen was unremarkable. The patient was scheduled to undergo EGD. During the EGD, granularity and mild congestion of the gastric mucosa were observed in the antrum (Fig. 1). Multiple biopsies were taken from the stomach and duodenum, and later pathologic analysis of the hematoxylin and eosin - and Giemsa-stained sections revealed mild chronic inflammation with focal activity, indicating the presence of H. pylori in the gastric antrum. Therefore, the patient was started on 20 mg of omeprazole daily, 500 mg of clarithromycin twice daily, and 500 mg of metronidazole twice daily for 15 days, followed by 20 mg of omeprazole therapy for 1 month; however, the patient’s symptoms remained the same.

**Table 1:** Complete laboratory report for the patient.

WBC	7.3 × 10^9^/L
Granulocytes	57.4%
Lymphocytes	36.5%
RBC	4.07 × 10^12/L
HGB	11.3 g/dL
HCT	32.8%
MCV	80.8 fL
PLT	389 × 10^9^/L
AST	20 U/L
ALT	6 U/L
Amylase	89 U/L
GGT	13 U/L

ALT = alanine aminotransferase, AST = aspartate aminotransferase, GGT = gamma-glutamyl transferase, HCT = hematocrit, HGB = hemoglobin, MCV = mean cell volume, PLT = platelets, RBC = red blood cells, WBC = white blood cells.

**Figure 2.**
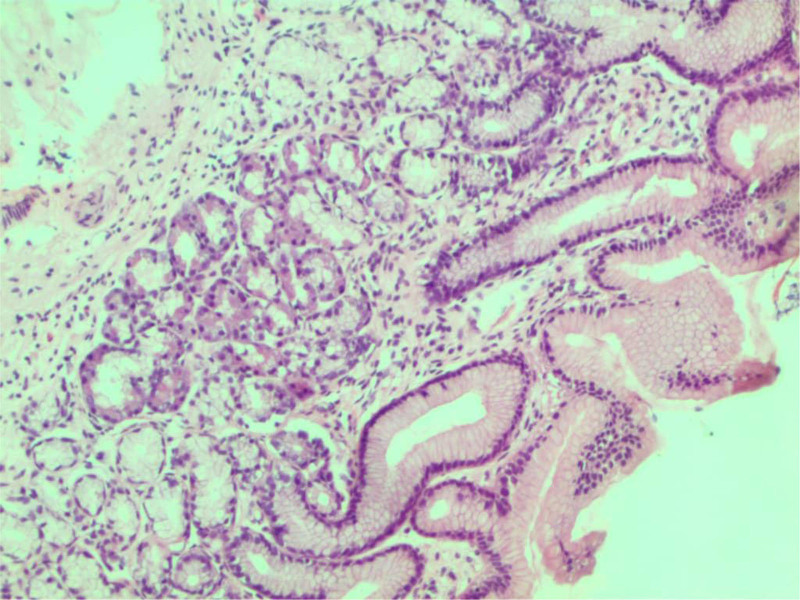
Lobules of pancreatic acinar cells in the gastric antrum.

Six months later, a second EGD was performed because of persistent pain, and biopsies were taken from the gastric corpus and antrum. The biopsies were negative for H. pylori, but antral biopsies showed lobules of cells, resembling pancreatic acinar cells, interspersed among the normal antral glands (Fig. 2). Moreover, lymphocytic aggregates were observed in the corpus biopsies. The fecal Helicobacter antigen test result was negative. Based on the histological findings, the patient was diagnosed with PACM. She was given 20 mg of omeprazole and 20 mg of domperidone thrice per day to control the symptoms; however, no significant improvement was achieved.

Due to the absence of an organic cause for the symptoms, the patient’s condition was suspected of being psychiatric in nature, so she was transferred to a psychiatric clinic for behavioral therapy, to no avail. One year after the initial presentation, the patient underwent a third EGD with biopsies that also showed PACM and absence of H. pylori. The clinical condition remained the same, with occasional abdominal pain, nausea, and decreased appetite.

## 3. Discussion and conclusions

PACM of the gastric mucosa is a condition characterized by the presence of clusters of pancreatic-like cells in the stomach, without a neuroendocrine component (islets of Langerhans).^[1]^ After a comprehensive review of the literature, it was found that only 16 cases of PACM of the stomach in children have been reported (Articles 2 and 3 were written by the same authors and in an overlapping time period, so the actual number of cases might be 10).^[2,3,5]^ This condition has also been observed in the liver and gastroesophageal junction.^[1,6]^ In a study conducted in 1993, Doglioni et al estimated that PACM was found in 1% of gastric biopsies and 12% of gastrectomy specimens, with no significant difference with respect to age or sex.^[1]^ Various studies have reported an association between PACM and both H. pylori and chronic atrophic gastritis.^[1,4]^ It has also been shown to occur more frequently in the antrum than in other parts of the stomach, which could be explained by the fact that gastritis in H. pylori tends to occur in the antrum.^[4]^ PACM occurring in the fundus is almost always accompanied by atrophic gastritis.^[1]^ In children, however, there have been some reported cases without gastritis or H. pylori infection, which implies that PACM could also occur due to developmental errors of the gastric mucosa.^[3]^ Studies conducted on rats have shown that PACM can develop after omeprazole therapy.^[7]^ In our patient, PACM was not present in the first EGD when H. Pylori was diagnosed; however, subsequent EGDs following treatment with triple therapy showed PACM.

The specific mechanism by which the metaplasia occurs remains unknown. Doglioni et al showed that pancreatic cells nestled around gastric glands share the basement membrane of the normal cells and are connected to them using desmosomes, which implies that these pancreatic cells developed as a result of metaplasia of normal gastric glands.^[1]^ In addition, PDX-1, a transcription factor important for the normal development of pancreatic cells, was upregulated in metaplastic cells and their neighboring gastric parietal cells, in contrast to normal parietal cells, where PDX-1 expression was not detected, implying that parietal cells could be the progenitor cells of the metaplasia.^[8]^ Another possible explanation for this condition is that the cells of the stomach, pancreas, liver, gallbladder, and intrahepatic bile ducts share the same embryonic origin, making the transformational process an error of differentiation into proper cells.^[1]^

PACM might be confused with pancreatic heterotropia since both conditions result in pancreatic cells in the stomach; however, the pancreatic cells in PACM form nests around gastric glands in the mucosa, whereas in pancreatic heterotropia, the cells are typically located in the submucosa or the outer muscular layer. Furthermore, pancreatic cells in PACM do not form islets of Langerhans and do not display immunoreactivity to any pancreatic hormones, in contrast to the pancreatic cells in pancreatic heterotropia.^[1]^ Furthermore, unlike intestinal metaplasia of the stomach, PACM is not considered a precancerous lesion.

Along with this case report, there are around 15 documented cases of PACM of the stomach in children. This article is important because it sheds light on a condition that is often overlooked or underdiagnosed owing to its rarity in this age group and uncertainty regarding clinical significance. It is important to take notice of this kind of metaplasia in gastric biopsies, especially among children, in order to assess the progression of the condition as the patients grow older. More research is required to understand the long-term complications to gauge the need for definite treatment.

## Author contributions

**Writing – original draft:** Muhsen Issa, Wafaa Taleb, Rana Issa.

**Writing – review & editing:** Muhsen Issa, Wafaa Taleb, Rana Issa.

**Conceptualization:** Ali Ibrahim, Rana Issa.

**Supervision:** Ali Ibrahim, Rana Issa.
